# mTOR pathway mediates endoplasmic reticulum stress-induced CD4^+^ T cell apoptosis in septic mice

**DOI:** 10.1007/s10495-022-01740-1

**Published:** 2022-06-27

**Authors:** Guangxu Bai, Hao Wang, Na Cui

**Affiliations:** 1 Department of Critical Care Medicine, Peking Union Medical College Hospital, Peking Union Medical College and Chinese Academy of Medical Science, Beijing, 100730 China; 2 Department of Critical Care Medicine, Beijing Jishuitan Hospital, Beijing, 100035 China; 3 Department of Clinical Laboratory, Peking Union Medical College Hospital, Peking Union Medical College, Chinese Academy of Medical Science; Beijing Key Laboratory for Mechanisms Research and Precision Diagnosis of Invasive Fungal Diseases, Beijing, 100730 China

**Keywords:** Endoplasmic reticulum stress, Apoptosis, CLP, IRE1–JNK, Mammalian target of rapamycin

## Abstract

Endoplasmic reticulum stress (ERS) has been well documented to participate in the pathophysiological processes of apoptosis in many diseases. Inhibition of ERS ameliorates pathological organ injury. However, the upstream signaling pathways and molecular regulatory mechanisms of which are still unknown. mTOR, an evolutionarily conserved protein kinase, is a key regulator of apoptosis. Hence, in this study, a classical cecal ligation and puncture (CLP) sepsis model was constructed by using the T cell-specific knockout *mTOR* and *TSC1* (Tuberous Sclerosis Complex, the inhibitor of mTOR signaling pathway) mice to explore the underlying signaling pathway and molecular mechanism of host immune imbalance caused by apoptosis in sepsis. We found that mTOR may modulate septic T cell apoptosis by regulating Akt–IRE1–JNK pathway. To further clarify the possible mechanism, the specific inhibitors of PI3K-Akt and IRE1–JNK were used to intervene in mice before/after CLP, respectively. By analyzing the proteins of mTOR-ERS signaling pathway and the expression of apoptosis-related proteins and genes, we found that mTOR mediated the ER stress induced CD4^+^ T cell apoptosis in Septic mice by negatively regulating the Akt–IRE1–JNK-Caspase 3 signaling cascades. These results indicate that mTOR–Akt–IRE1α–JNK signaling pathway mediated the Endoplasmic reticulum stress induced CD4^+^ T cell apoptosis in Septic mice.

**Supplementary Information:**

The online version contains supplementary material available at 10.1007/s10495-022-01740-1.

## Introduction

With the profound understanding of the pathophysiology of sepsis, recent decades has seen an increasing body of evidence supporting the central role of the immune system in sepsis. However, our current knowledge of the mechanism by which sepsis affects immunity and vice versa remains limited [1]. T cell immunity, indispensable to adaptive immunity, is critical in the occurrence and development of sepsis. Many studies [2–4] have shown that apoptosis is an important cause of the decrease in T cells associated with sepsis, while the contributing factors of apoptosis are complex [5, 6], including internal factors such as endoplasmic reticulum stress (ERS), mitochondrial p53, and external factors such as FAS. Under different environments, many pathways are intertwined. Thus, how to disentangle these factors and promote the transformation of pre-clinical basic research results into clinical treatment methods has become an urgent problem.

Cellular dysfunction characterized by ERS was recently proposed as an important factor causing T cell apoptosis in severe infections and leading to immune dysfunction [7]. ERS refers to the accumulation of a large number of unfolded or misfolded proteins in pathological states such as sepsis, trauma, and ischemia, resulting in the loss of endoplasmic reticulum homeostasis [8]. In this case, cells may activate a self-defense mechanism, the unfolded protein response (UPR), that responds to the stress through inositol-requiring enzyme 1 (IRE1), double-stranded RNA-dependent protein kinase-like endoplasmic reticulum kinase (PERK), and transcriptional activator 6 (ATF6), then reduces unfolded and misfolded protein accumulation in the endoplasmic reticulum via different signaling cascades to restore intracellular homeostasis [9]. However, this defense mechanism may be ineffective or inadequately responsive in a rapidly progressive state of critical illness, activating programmed cell death including apoptosis and autophagy when the UPR fails to address protein accumulation, referred to as ER stress pathway-apoptosis (ER stress-apoptosis) [10, 11]. Since studies have demonstrated that ERS induces apoptosis mainly through IRE1 and PERK signaling by increasing the expression of C/EBP homologous protein (CHOP) and the activation of cleaved proapoptotic factor caspase-3 [12] rather than through ATF6 [13, 14], IRE1 and PERK may be the key signaling pathway regulating the ER stress-apoptosis of T cells during sepsis.

As an evolutionarily conserved protein kinase, mTOR coordinates protein synthesis and participates in regulating cell proliferation and metabolism by integrating external environmental elements and internal signals [15]. It is irreplaceable in controlling and shaping various functions of innate and adaptive immune cells, especially in the differentiation, survival, and metabolic reprogramming of T cell subsets, and is the main regulator determining the type and fate of immune cell differentiation [16]. Furthermore, mTOR is closely related to ERS: mTORC1, an mTOR complex, regulates apoptosis in drug-induced ERS by inhibiting Akt expression and selectively activating the IRE1–JNK pathway [17, 18]. TSC1 as an inhibitor of mTOR signaling pathway is one of the Tuberous Sclerosis Complex has also been shown that plays a key role in the regulation of apoptosis and protein synthesis [19].

Hence, we hypothesized that mTOR likely affects apoptosis by regulating T cell ERS during sepsis. To verify this hypothesis, we used T cell-specific knockout *mTOR* and *TSC1* mice to create sepsis models using the classical CLP method to observe the effects of mTOR on ERS and the apoptosis of mice CD4^+^ T cells and the potential molecular regulatory mechanism.

## Materials and methods

### Mice

Healthy male C57BL/6 N mice aged 6–8 weeks and weighing 20–25 g were used in this study and were purchased from the Animal Laboratory Center of Peking Union Medical College Hospital (Beijing, China). *mTOR*^*loxp/loxp*^, *TSC1*^*loxp/loxp*^ and *Lck-Cre* mice were a generous gift from Dr. Yong Zhao (State Key Laboratory of Biomembrane and Membrane Biotechnology, Institute of Zoology, Chinese Academy of Sciences, Beijing, China). T cell-specific *Mtor* and *Tsc1 *gene knockout mice were constructed as described in a previous study [20] to inhibit/enhance the activity of the mTOR pathway in T cells. Mice were randomly divided into seven groups: WT+SHAM, WT+CLP, WT+CLP+4-PBA (4-PBA, an inhibitor of ERS), mTOR-CKO (conditional knockout) +CLP, TSC1-CKO + CLP, mTOR-CKO+CLP+LY294002 (LY294002, an inhibitor of the PI3K–Akt pathway), and TSC1-CKO+CLP+SP600125 (SP600125, an inhibitor of the IRE1–JNK pathway) with six mice in each. All mice were subjected to a 12 h day/night cycle for at least 1 week under specific aseptic conditions before the experiment.

### Model and reagents

**Fig. 1:**
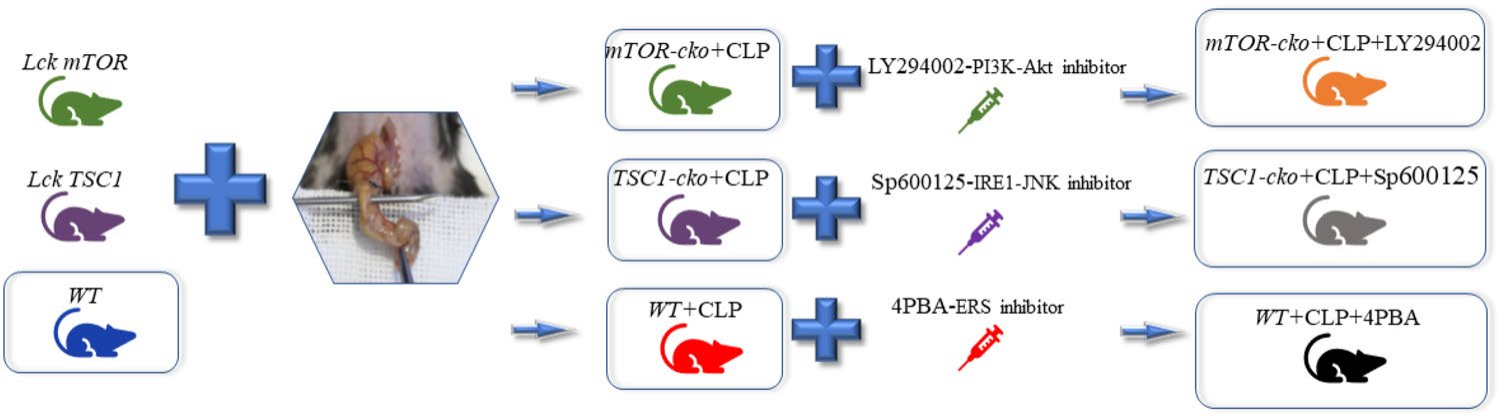
The flow chart of this research. Mice were randomly divided into seven groups: WT+SHAM, WT+CLP, WT + CLP+4-PBA (4-PBA, an inhibitor of ERS), mTOR-CKO (conditional knockout) + CLP, TSC1-CKO+CLP, mTOR-CKO+CLP+LY294002 (LY294002, an inhibitor of the PI3K–AKT pathway), and TSC1-CKO+CLP+SP600125 (SP600125, an inhibitor of the IRE1–JNK pathway) with six mice in each

In this study, lethal septic shock model mice were induced by the classical CLP procedure. The protocol was performed as previously described [21]. Briefly, mice were adequately anesthetized with 10% chloral hydrate (0.3 ml/100 g body weight, intraperitoneal injection). A 1.5 cm incision was made in the middle of the abdomen to expose the cecum, followed by ligation at 3/4 of the distal cecum. Next, cecal puncture was performed twice with a 22G needle and a small amount of feces was forced out to flow into the abdominal cavity. Sham-treated mice were subjected to the same laparotomy without CLP. All mice were immediately administered fluid resuscitation after abdominal closure [22]. Mice of the 4-PBA (T1535 CAS 1716-12-7; Topscience) intervention group were intraperitoneally injected with 40 mg/kg 4-PBA 1 h before CLP [23]. Mice of the LY294002 (T2008 CAS 154447-36-6; Topscience) group were intraperitoneally injected with 40 mg/kg LY294002 immediately after CLP [24] and the SP600125 (T3109 CAS 129-56-6; Topscience) intervention group was intraperitoneally injected with 15 mg/kg SP600125 at 1 h after surgery [25]. The flow chart with a diagram of the groups of mice studied was shown as Fig. 1.

### Splenic lymphocyte isolation and CD4^+^***T cell counting***

All mice were sacrificed 12 h after CLP modeling and spleens were surgically removed. Lymphocytes from the spleens were isolated according to the instructions of the mouse splenic lymphocyte isolation kit (P8860; Solarbio). Next, 90 μL PBS buffer containing 5% serum was added to the obtained spleen cell suspension for resuspension, and 10 μL magnetic beads (L3T4 MicroBeads; Miltenyi Biotec) coupled with CD4^+^ T cell antibody were then added, incubated at 4 °C for 20 min, and washed. After resuspending again, CD4^+^ T cells were isolated by negative selection using separation columns and centrifuged in RPMI-1640 medium, and their viability and number was quantified by trypan blue exclusion with a TC20 automated cell counter (Bio Rad).

### Transmission electron microscopy

First, isolated CD4^+^ T cells from each group were fixed by 2.5% glutaraldehyde in sodium phosphate buffer (0.1 M, pH 7.3) at 4 °C overnight, post-fixed with 1% osmium at 4 °C for 3 h, dehydrated via graded ethanol, embedded into blocks using a Spurr Embedding Kit (Spi-Chem), and polymerized at 70 °C. Then, the embedded blocks were cut into 70 nm slices on an ultramicrotome (Leica EM UC6). The ultrathin sections were stained with aqueous uranyl acetate and lead citrate and observed under a transmission electron microscope (JEM1230; Jeol).

### Western blotting

Western blotting analysis was performed as described previously [24]. The antibodies used in this study were all purchased from Affinity Biosciences, as follows: anti-GRP78 (Cat# AF5366), anti-DDIT/CHOP (Cat# DF6025), anti-mTOR (Cat# AF6308), anti-phospho(P)-mTOR (Cat# AF3308), anti-P-p70S6 (Cat# AF6226), anti-P-p70S6 kinase (Thr389/Thr412) (Cat# AF3228), anti-pan-Akt1/2/3 (Cat# AF6261), anti-P-pan-Akt1/2/3 (Ser473) (Cat# AF0016), anti-P-IRE1 (Ser724) (Cat# AF7150), anti-JNK1/2/3 (Cat# AF6318), anti-P-JNK1/2/3 (Thr183+Tyr185) (Cat# AF3318), anti-caspase-3 (Cat# AF6311), and anti-BCL-2 (Cat# AF6139) antibodies. Anti-beta actin (β-actin) (Cat# AF7018) and anti-COX IV (Cat# AF5468) antibodies were used as loading controls.

### Apoptosis assay

The apoptosis of CD4^+^ T cells was detected by Annexin V/FITC Detection Kit (BD) and propidium iodide (PI) (BD) staining. FACSCalibur (BD) and Cell Quest Pro software were used to examine stained cells at an excitation wavelength of 488 nm and an emission wavelength of 530 nm. FlowJo software was used to analyze the sample and illustrate the result as a dot plot.

### Real-time quantitative PCR for BIM

In this study, SYBR Green Real-Time PCR Master Mix was used to detect the transcription level of *BIM* mRNA in the isolated CD4^+^ T cells. *BIM* mRNA as a member of the famous Bcl-2 protein family, is one of the most active pro-apoptotic proteins and has been widely used in the detection of cell apoptosis level [26]. In this research *BIM* mRNA was examined using the following primers: 5ʹ-GAGATACGGATTGCACAGGA-3ʹ and 5ʹ-TCAGCCTCGCGGTAATCATT-3ʹ. *ACTB* was used as an internal control. The fold changes were calculated by relative quantification (2^−△△Ct^).

### Statistical analysis

Data were analyzed using SPSS version 24.0 software (SPSS Inc., Armonk, NY, USA). All data for continuous variables in this study had normal distributions and are shown as the mean ± standard deviation (SD). Statistical analysis was performed by two-tailed Student’s t-test or one-way analysis of variance (ANOVA) followed by Dunn’s/Tukey’s test to compare data in different groups. P < 0.05 was considered statistically significant.

## Results

### Target gene knockout confirmed by mRNA expression levels in LCK–mTOR and LCK–TSC1 mice

In this study, we used *mTOR*^*loxp/loxp*^, *TSC1*^*loxp/loxp*^ and *Lck-Cre* mice for the construction of T cell-specific knockout *mTOR* and *TSC1* mice to inhibit and enhance their mTOR pathway, respectively. Lck refers to lymphocyte protein tyrosine kinase, which has an ability to mark target genes in T lymphocytes through the loxp-cre system and acts as a promoter to knockout the T cell-specific target gene by backcross [27]. The mRNA expression levels of *mTOR* and *TSC1* in mice were detected by RT-PCR to confirm knockout of the target genes, which can be found in supplementary material (Fig. S1).

### ERS induced apoptosis of CD4^+^***T cells in sepsis***

**Fig. 2:**
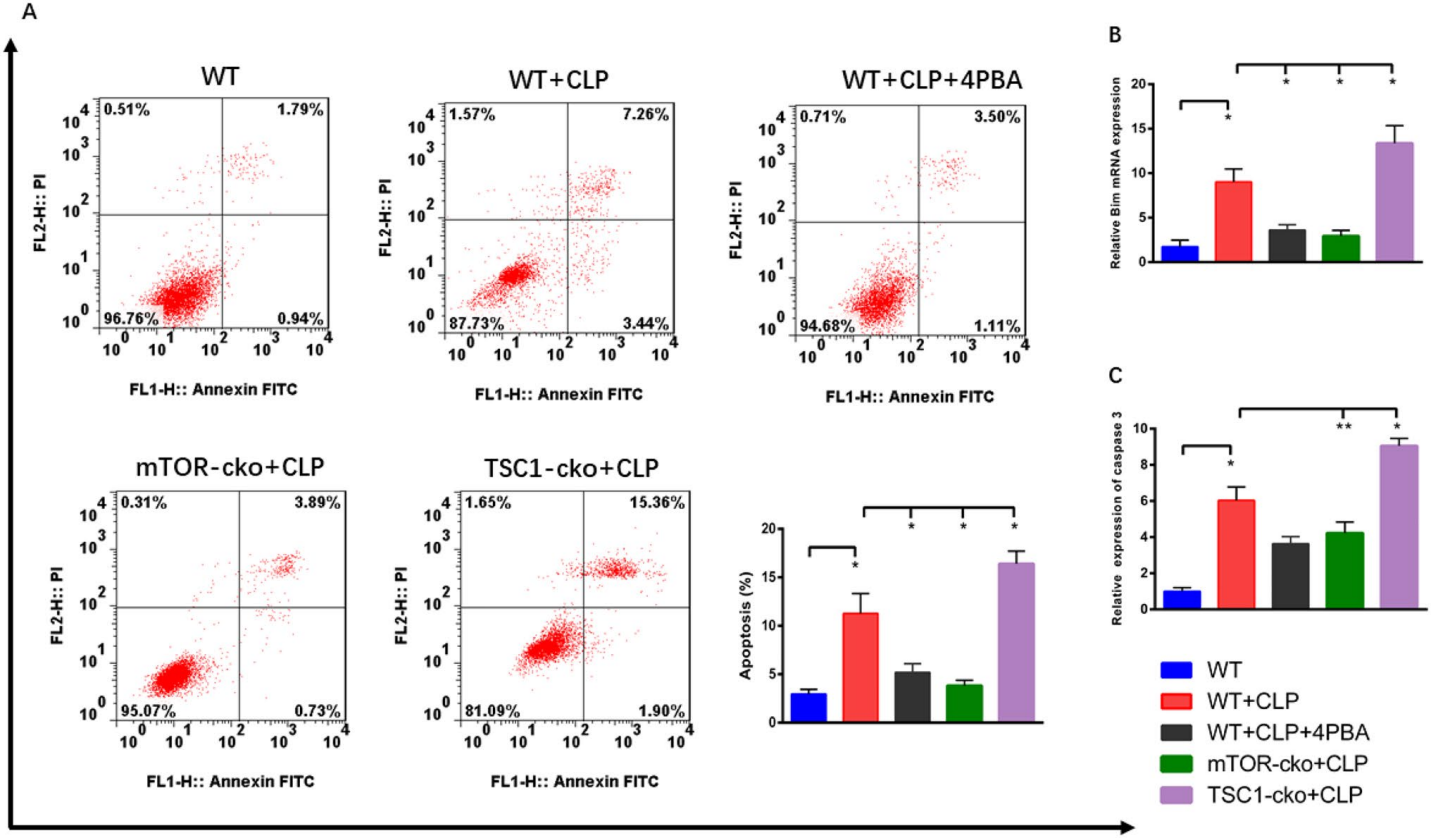
Evaluation of apoptosis in CD4^+^ T cells in sepsis. The subgroup of annexin-V-positive and PI-negative CD4^+^ T cells was considered to be undergoing early apoptosis, while the annexin-V-positive and PI-positive CD4^+^ T cells were considered to be undergoing late apoptosis. The apoptosis rate of CD4^+^ T cells, as analyzed by the ratio of annexin-V-positive and PI-positive/-negative CD4^+^ T cells (**A**). Relative RNA expression of the pro-apoptotic gene *BIM* (**B**). Relative protein expression of the pro-apoptotic protein caspase-3 (**C**). Data are shown as Mean ± SD (n = 6). Statistically significant differences were determined by one-way analysis of variance (ANOVA) followed by Dunn’s/Tukey’s test, ∗P < 0.05, ∗∗P < 0.01

To confirm that ERS induced apoptosis of CD4^+^ T cells in sepsis, we first compared the percentage of apoptosis in splenic CD4^+^ T cells and the expression of the pro-apoptotic gene *BIM* and expression of the pro-apoptotic protein caspase-3 between WT+SHAM and WT+CLP mice. As shown in Fig. 2, compared with the WT+SHAM group, the apoptosis of CD4^+^ T cells (Fig. 2A) and the expression of *BIM* and caspase-3 were increased in the WT+CLP group (Fig. 2B, C). We next compared the above data between WT+CLP mice with and without 4-PBA intervention and found that WT+CLP mice with 4-PBA intervention had fewer apoptotic CD4^+^ T cells and lower expression of *BIM* than the WT+CLP group. In addition, we also found that compared with the WT+CLP group, mice in the mTOR-CKO+CLP group had a lower proportion of apoptotic CD4^+^ T cells and lower *BIM* and caspase-3 expression, while the data in the TSC1-CKO+CLP group showed a contrary trend.

### Swollen endoplasmic reticulum was observed in the subcellular structure of T cells during sepsis

**Fig. 3:**
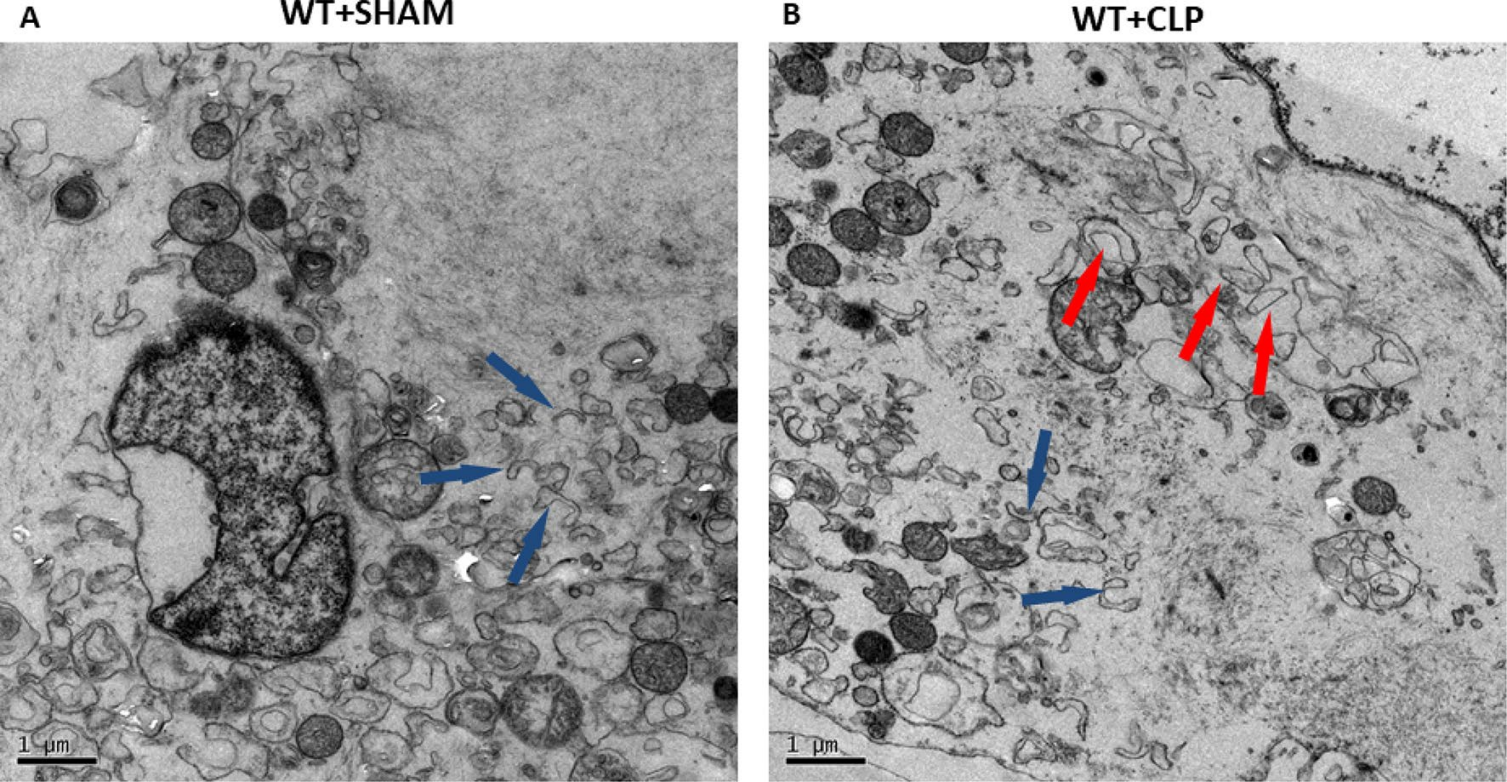
Electron microscopy images showing the ultrastructure of CD4^+^ T cells in septic mice. Ultrastructural features of endoplasmic reticulum in CD4^+^ T cells. The process of ERS was investigated by TEM. In WT+SHAM group mice (**A**), CD4^+^ T cells were normal in appearance, revealing normal endoplasmic reticulum (blue arrow) in the cytosol. WT+CLP group mice (**B**) displayed not only normal (blue arrow) but also swollen endoplasmic reticulum (red arrow) with three mice in each

To further confirm the existence of ERS in CD4^+^ T cells during sepsis, we observed the ultrastructure of splenic CD4^+^ T cells by transmission electron microscopy (TEM). Figure 3A shows that the splenic CD4^+^ T cells of the WT+SHAM group had normal endoplasmic reticulum (blue arrow). Figure 3B shows the swollen endoplasmic reticulum (red arrow) in CD4^+^ T cells of the WT+CLP group.

### ERS induced by sepsis activates the mTOR signaling pathway

**Fig. 4:**
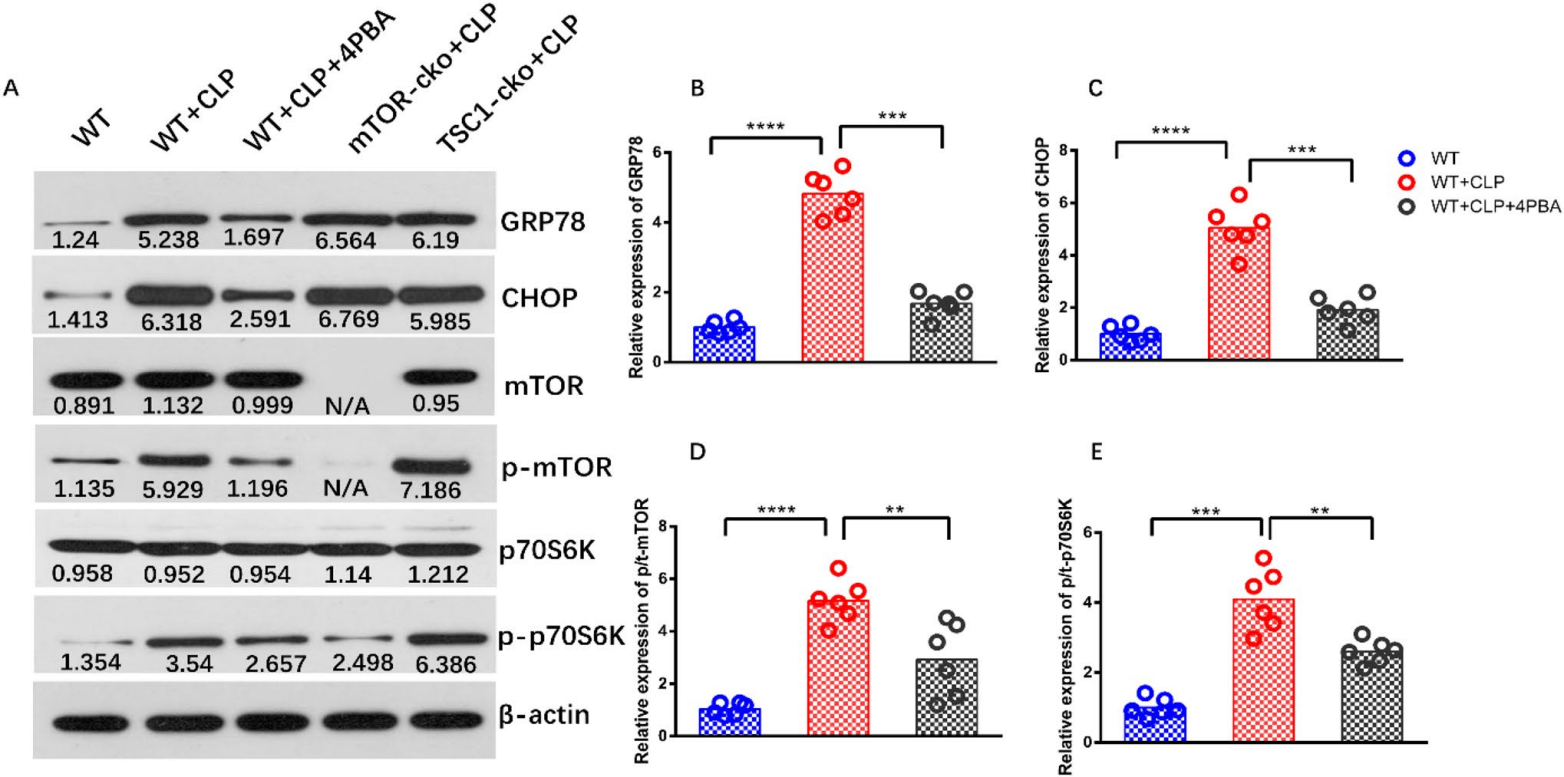
Expression of ERS-UPR- and mTOR-related proteins in splenic CD4^+^ T cells of septic mice. Protein expression of GRP78, CHOP, mTOR, p-mTOR, p70S6k, p-p70S6k (**A–E**) in CD4^+^ T cells were quantified by western blotting and showed as the relative expression values of β-actin, which was used as a loading control to normalize the protein levels. In order to highlight the activation level of p-mTOR and p-p70S6K in this signaling pathway, the ratio of p-mTOR to mTOR (p/t mTOR) and p-p70S6K to p70S6K (p/t p70S6K) were used for statistics. Data are shown as Mean ± SD (n = 6). Statistically significant differences were determined by two-tailed Student’s t-test. **P < 0.01, ***P < 0.001, ****P < 0.0001

The expression of ERS marker proteins GRP78 and CHOP and mTOR pathway marker proteins p-mTOR, p70S6K, and p-p70S6K were evaluated in this study to explore the potential regulatory mechanism of mTOR in the ERS induced by sepsis. As shown in Fig. 4, the levels of ERS marker proteins and mTOR pathway proteins in the WT+CLP group were significantly increased compared with the WT+SHAM group, while WT mice pre-treated with 4-PBA before CLP had lower expression of these proteins than WT+CLP mice.

### mTOR modulates the IRE1–JNK pathway by negatively regulating the expression of Akt

**Fig. 5:**
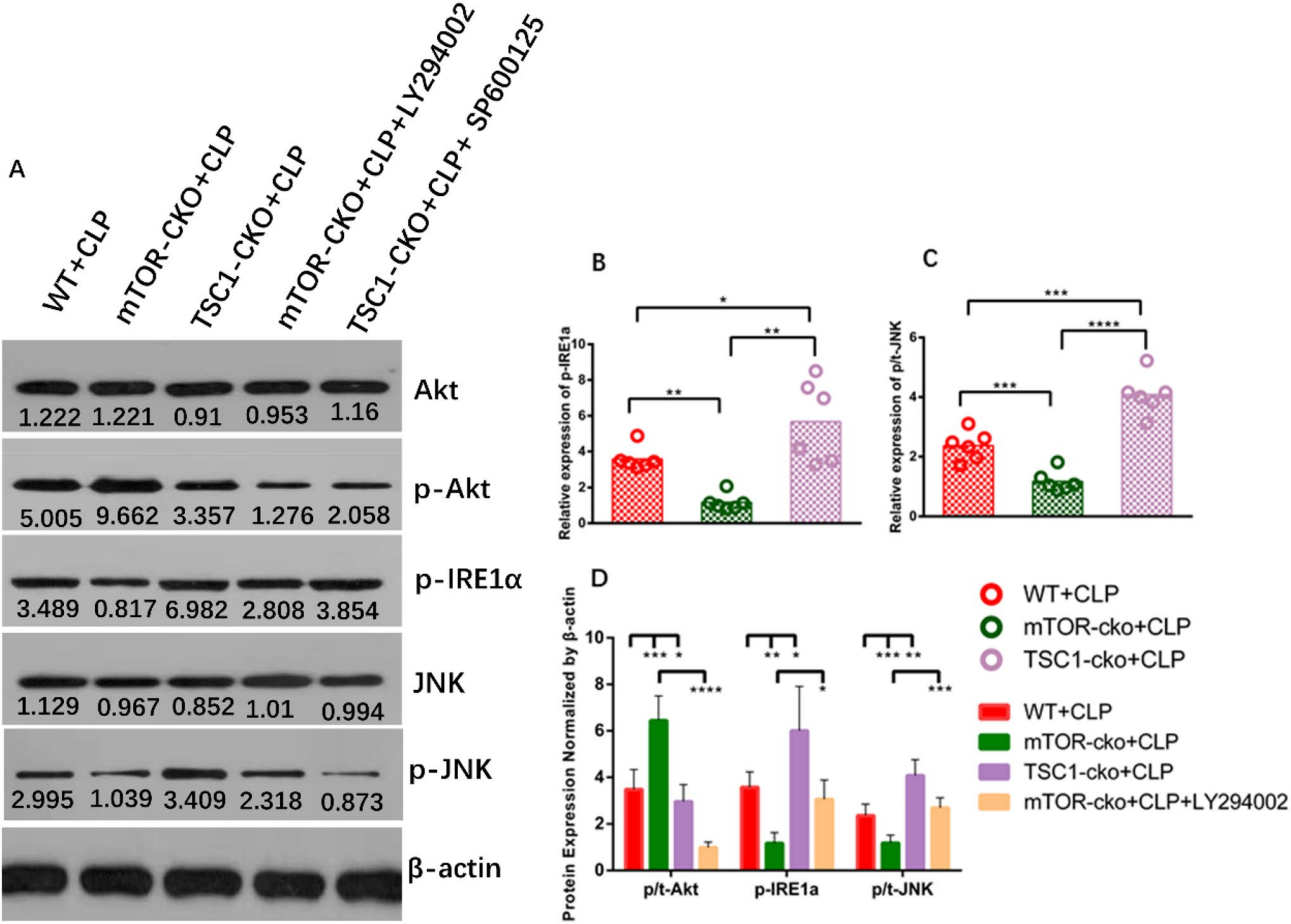
Expression of Akt and IRE1-JNK-related proteins in splenic CD4^+^ T cells of sepsis mice. Protein levels of Akt, p-Akt, p-IRE1α, JNK, and p-JNK (**A-D**) in CD4^+^ T cells were quantified by western blotting and showed as the relative expression values of β-actin, which was used as a loading control to normalize protein levels. For highlighting the activation level of p-Akt and p-JNK in this signaling pathway, the ratio of p-Akt to Akt (p/t Akt) and p-JNK to JNK (p/t JNK) were used for statistics. Data are shown as Mean ± SD (n = 6). Statistically significant differences were determined by one-way analysis of variance (ANOVA) followed by Dunn’s/Tukey’s test. *P < 0.05, **P < 0.01, ***P < 0.001, ****P < 0.0001

Previous studies [17, 18] have shown that mTOR may modulate ERS by negatively regulating Akt expression. Therefore, to further reveal whether the same role was played by mTOR in this process, we used T cell-specific *mTOR* and *TSC1* knockout mice for CLP modeling and analyzed the expression of mTOR downstream protein p-Akt and ERS pathway proteins P-IRE1α, JNK, and P-JNK. As shown in Fig. 5, compared with the WT+CLP group, the expression of IRE1–JNK pathway-related proteins in the mTOR-CKO+CLP group was significantly decreased, while they were increased in the TSC1-CKO+CLP group (Fig. 5A–C). Whereafter, to prove that mTOR was involved in the modulation of the IRE1–JNK pathway through the negative regulation of Akt, we administered a classical PI3K–Akt inhibitor LY294002 after CLP in mTOR-CKO mice as a control group. As we expected, compared with WT+CLP mice, the expression of the mTOR downstream protein p-Akt was increased in the mTOR-CKO+CLP group and decreased in the TSC1-CKO + CLP group. Mice in the mTOR-CKO+CLP+LY294002 group had higher expression of IRE1–JNK pathway-related proteins than mTOR-CKO+CLP group mice (Fig. 5A, D).

### The mTOR pathway mediated ERS-induced CD4^+^ T cell apoptosis in septic mice by regulating the IRE1–JNK pathway

**Fig. 6:**
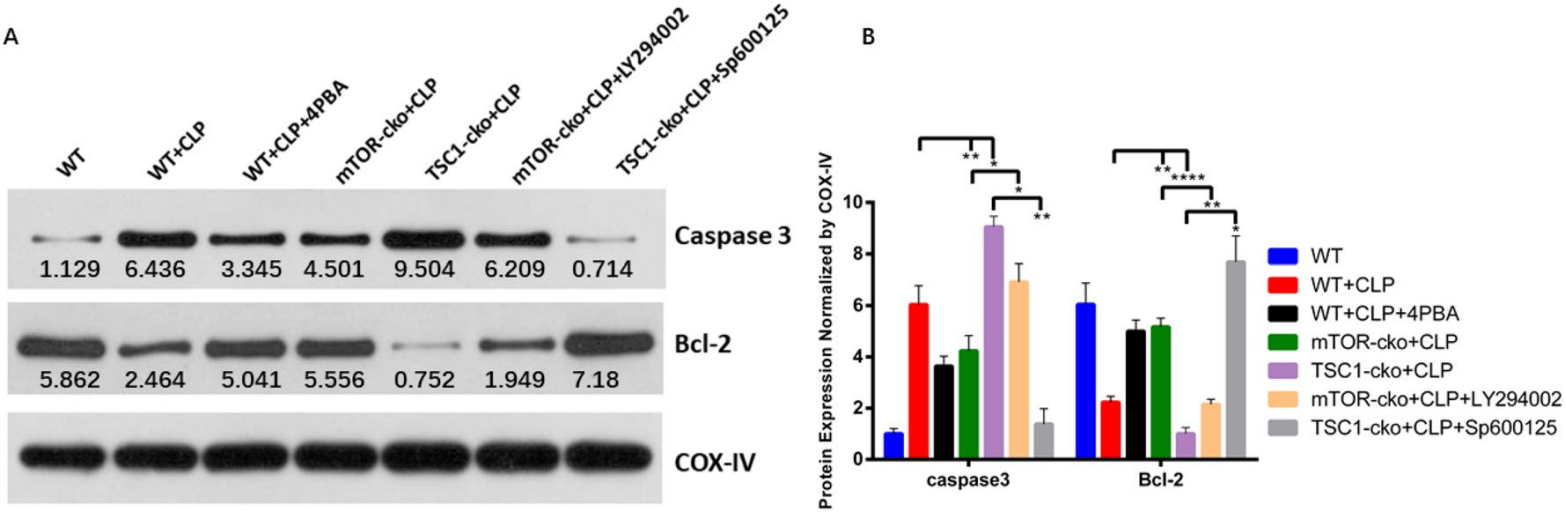
Levels of IRE1α-related pro-apoptotic protein caspase-3 and anti-apoptotic protein BCL-2. Expression of pro-apoptotic protein caspase-3 and anti-apoptotic protein BCL-2 (**A**, **B**) in CD4^+^ T cells. Protein expression were determined by western blotting and showed as the relative expression values of COX-IV. COX-IV was used as a loading control to normalize protein levels. Data are shown as Mean ± SD (n = 3). Statistically significant differences were determined by one-way analysis of variance (ANOVA) followed by Dunn’s/Tukey’s test. *P < 0.05, **P < 0.01, ****P < 0.0001

**Fig. 7:**
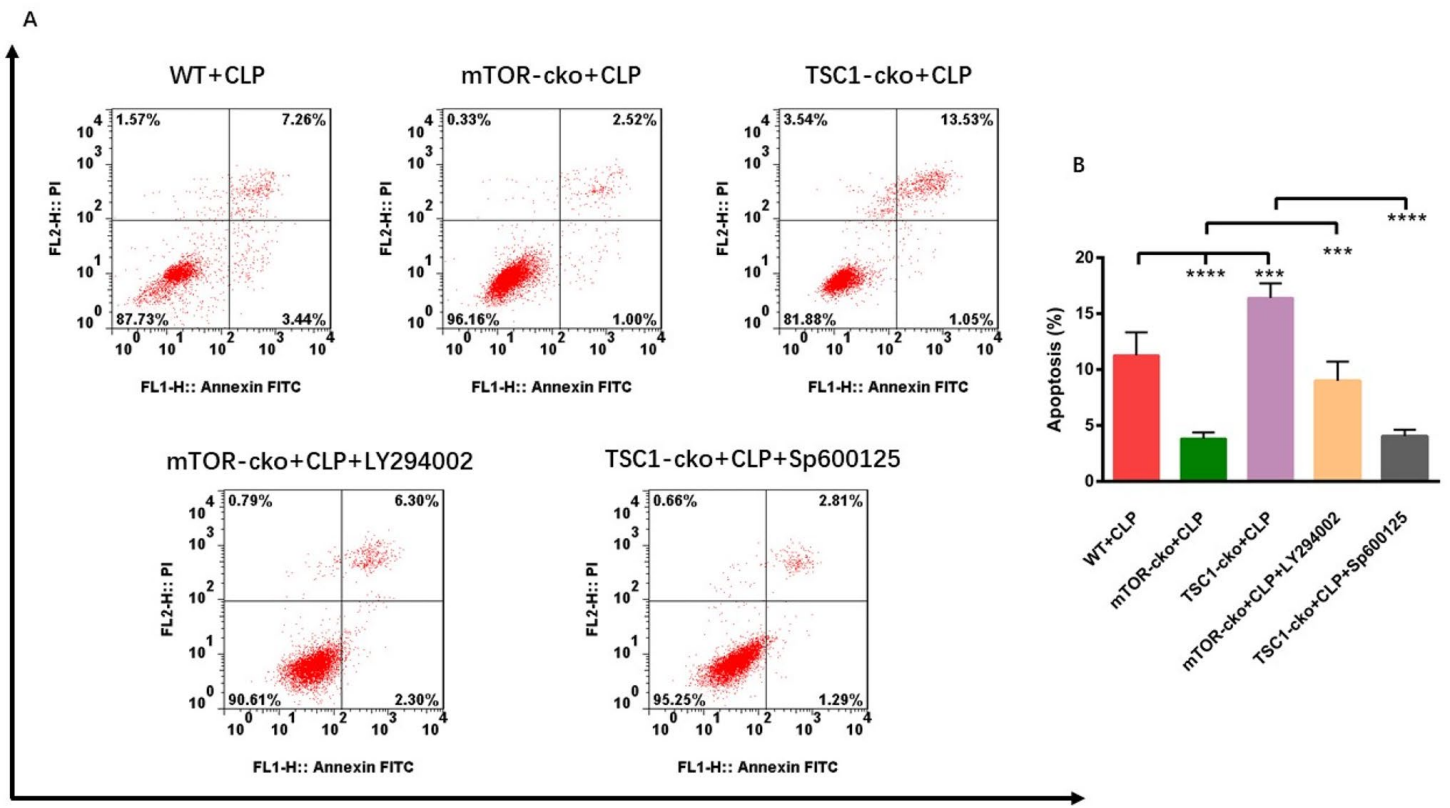
Comparison of apoptosis of CD4^+^ T cells in septic mice with or without inhibitor intervention. The subgroup of annexin-V-positive and PI-negative CD4^+^ T cells was considered to be undergoing early apoptosis, while annexin-V-positive and PI-positive CD4^+^ T cells were considered to be undergoing late apoptosis (**A**). The apoptosis rate of CD4^+^ T cells, as analyzed by the ratio of annexin-V-positive and PI-positive/-negative CD4^+^ T cells (**B**). Data are shown as Mean ± SD (n = 6). Statistically significant differences were determined by one-way analysis of variance (ANOVA) followed by Dunn’s/Tukey’s test. ****P < 0.0001

Finally, to verify that mTOR affects the apoptosis of CD4^+^ T cells in septic mice by regulating the ERS–IRE1–JNK pathway, we administered SP600125, an inhibitor of the IRE1–JNK pathway, to TSC1-CKO mice after CLP. Compared with TSC1-CKO + CLP mice, TSC1-CKO+CLP+SP600125 mice had lower expression of the pro-apoptotic protein caspase-3 and higher expression of the anti-apoptotic protein BCL-2 (Fig. 6). Figure 7 shows a comparison of the apoptosis of CD4^+^ T cells in these five groups. Both groups of mice that underwent inhibitor intervention had a statistically significant difference in the rate of CD4^+^ T cell apoptosis compared with their controls, which further supported that the mTOR pathway may mediate ERS-induced CD4^+^ T cell apoptosis in septic mice by regulating the ERS–IRE1α–JNK signaling pathway. In addition, study of Kato et al. [17] has confirmed that mTOR inhibits IRE1-JNK by inhibiting the expression of Akt and thus reduce cell apoptosis. Since IRE1-JNK may be the last step to activate apoptosis, we speculated that Sp600125 might also reduce apoptosis level in mTOR-cko CLP mice, but this result was not correlated with the signaling pathway in this study.

## Discussion

To the best of our knowledge, this is the first study to demonstrate that the mTOR pathway mediated ERS-induced CD4^+^ T cell apoptosis in septic mice by regulating the ERS–IRE1α–JNK signaling pathway. Our results suggest that the mTOR–Akt–IRE1α–JNK signaling pathway may be a promising new target for clinical immunotherapy of sepsis with great research potential and value, providing a new direction for the immunotherapy of sepsis.

The Third International Consensus Definitions for Sepsis and Septic Shock (Sepsis-3) [28] indicates that host immune imbalance is the core mechanism of lethal organ dysfunction in sepsis patients. Studies [2, 3] have shown that persistent lymphocytopenia induced by apoptosis is closely related to the occurrence of host immune imbalance and is an independent risk factor for the poor prognosis of sepsis patients. The study of the apoptosis, autophagy, and function of T cells, as well as the analysis of the interaction between infection and host immunity from the perspective of T cells, may be a breakthrough in clinical research on sepsis patients, and a bridge between clinical and basic sepsis research. Thus, an increasing number of researchers [29, 30] are focusing on the signaling regulatory mechanism of lymphocyte apoptosis in patients with sepsis, hoping to improve host immune dysfunction in patients with sepsis by targeting this pathway.

Under normal conditions, GRP78/Bip forms a stable complex with transmembrane proteins (ERS receptors) on the surface of the endoplasmic reticulum and participates in protein folding as a molecular chaperone [14]. However, ERS caused by many pathological conditions results in separation of GRP78/Bip from these transmembrane proteins, which are then activated to sense ERS and initiate the UPR [9]. Thus, the expression level of GRP78 could be used to demonstrate the occurrence of ERS [31]. In this study, the splenic CD4^+^ T cells of WT+CLP mice showed swollen endoplasmic reticulum under transmission electron microscopy (Fig. 3B). The percentage of apoptotic CD4^+^ T cells and the expression of GRP78 and ERS-related apoptotic protein CHOP [32, 33] (Fig. 4A–C) in this group were both higher than in the WT+SHAM group. Compared with WT+CLP mice, the data above and the expression of the pro-apoptotic gene *BIM* in mice treated with ERS-specific inhibitor 4-PBA before CLP were significantly decreased (Fig. 2). These results suggested that ERS–UPR occurs in CD4^+^ T cells and affects the apoptosis of CD4^+^ T cells during sepsis. This is consistent with the study of Ma et al. [5] to some extent, which demonstrated that ERS is an important factor leading to abnormal apoptosis of lymphocytes in mice with sepsis. However, they discovered this phenomenon without an in-depth exploration of the potential mechanism and molecular signaling pathway.

mTOR is a serine/threonine protein kinase of the PI3K-related kinase family, which plays a cornerstone role in the physiological activities of cells and organisms by forming complex and interactive biological signaling networks to coordinate cell proliferation and environmental clues [15]. mTOR is closely associated with ERS. Kapuy [33] reported that there is a bidirectional crosstalk between mTOR and ERS, which is used to regulate ERS and programmed cell death. However, the mechanism was not described in their study. In the research of Kato [17] and Ozcan [18], they used drugs to induce ERS and found that mTORC1 may specifically activate IRE1 signaling cascades. On the basis of the above results, western blotting was first used in this study to detect the expression levels of mTOR pathway proteins in WT+SHAM and WT+CLP mice. It was found that P/total-mTOR and P/total-P70S6K in WT + CLP mice were significantly increased compared with levels in WT+SHAM mice, while mTOR-related protein expression in WT+CLP mice with 4-PBA intervention was significantly decreased compared with WT+CLP mice (Fig. 4), suggesting that mTOR is activated and involved in ERS caused by sepsis. Subsequently, the CLP model was constructed by T cell-specific knockout of *mTOR/TSC1*. By comparing the expression of downstream proteins in three ERS signaling cascades, we found that only the expression levels of downstream proteins P/total-IRE1 α and P/total-JNK of the IER1 signaling pathway showed significant differences (Fig. 5), suggesting that mTOR is not involved in the regulation of PERK–CHOP and ATF6–GRP78 signaling, but specifically regulates ERS through the modulation of the IRE1 signaling cascade. This result is consistent with the work of Alvarez et al. [34], who used drugs to induce ERS in vitro and found that mTOR specifically regulates IRE1 but not the AFT6 or PERK pathways to intervene in the apoptosis induced by ERS. In the recent study of Li et al. [35], mTOR was also demonstrated to be involved in the regulation of apoptosis in the process of osteoarthritis through the IRE1 signaling pathway. However, they also found that the mTOR–PERK–CHOP signaling pathway was also involved in the regulation of apoptosis induced by ERS, which was different from our results. We believe that one of the reasons for the different results is that their model simulates the pathophysiological process of osteoarthritis, which is much slower than that of sepsis with rapid progression and poor prognosis, and there may be some crosstalk between other signaling pathways. We believe that another possible reason for the different results between the two studies is that the IRE1 signaling pathway plays a dominant role in the process of ERS-induced apoptosis. This idea was confirmed by Upton [36] and Han [37]: IRE1α is necessary and sufficient for triggering apoptosis, while PERK and ATF6 are not necessary for activating cell apoptosis.

**Fig. 8:**
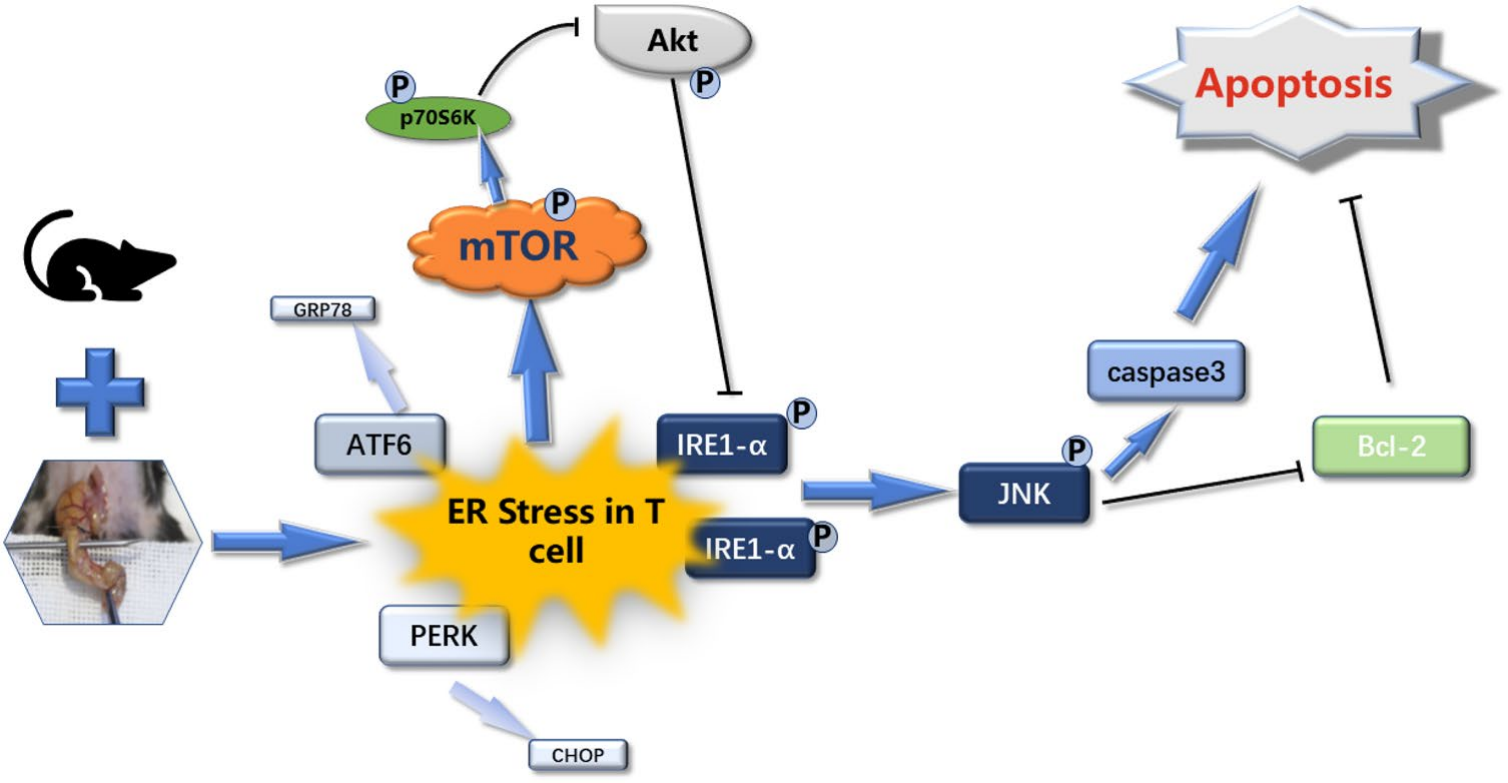
mTOR mediated ERS-induced CD4^+^ T cell apoptosis in septic mice by negatively regulating the Akt–IRE1–JNK–caspase 3 signaling cascade

To further clarify the complete molecular pathway of this signaling cascade, we used the classical PI3K–Akt inhibitor LY294002 to inhibit the activity of the Akt pathway in mTOR-CKO+CLP mice after CLP. As we expected, mTOR may modulate the IRE1–JNK signaling cascade through negative regulation of Akt (Fig. 5). In addition, through the administration of the IRE1A–JNK inhibitor SP600125 after CLP in TSC1-CKO mice, we found that mTOR selectively regulates IRE1α–JNK signaling and an important receptor in ERS–UPR through negative regulation of downstream Akt to influence apoptosis (Fig. 6). A comparison of apoptosis of CD4^+^ T cells in these five groups is shown in Fig. 7, which further supports the above results. Similarly, this result is also consistent with the in vitro cell research by Alvarez et al. in 2017 [34], whereby inhibition of mTOR–Akt activity improved IRE1-induced apoptosis and inflammatory injury. Figure 8 summarizes our current findings.

However, this study also had limitations. Because the purpose of this study was to prove the role of mTOR in regulating T cell apoptosis induced by ERS in sepsis, a longitudinal study at multiple time points throughout sepsis was not conducted; thus the dynamic influence of this mechanism on host immunity in the process of sepsis could not be determined.

Substantial evidence suggests that ERS is involved in various diseases such as sepsis, neurodegenerative diseases, cancer, and diabetes [38–40]. Inhibition of T cell ERS by drug or gene therapy strategies has been demonstrated to reduce the pathological features of experimental models of autoimmune diseases and tumors [41–44]. Some studies [45, 46] further found that the ERS component GRP94 CHOP was significantly increased in the process of myocardial inhibition in septic rats, and inhibition of ERS could protect against septic myocardial injury. Heme oxygenase-1 activation may attenuate sepsis-induced ERS and acute lung injury [47]. This suggested that ERS is a potential new target for clinical treatment of multiple organ failure and immunotherapy in sepsis. However, studies on ERS are limited to demonstrating its involvement in the pathophysiological process of various diseases, and few studies on its upstream regulatory pathways and molecular regulatory mechanisms exists. Our study showed the regulatory effect of mTOR–Akt on ERS–UPR in a murine CLP model by analyzing the proteins of the mTOR–Akt–ERS signaling pathway, and suggested that mTOR–Akt–IRE1α–JNK signaling has the potential to serve as a clinical immunotherapy target for sepsis and provide a new direction for sepsis immunotherapy.

## Supplementary Information

Below is the link to the electronic supplementary material.

Supplementary file1 (DOCX 1439 kb)

## Data Availability

The data that support the findings of this study are available from the corresponding author upon reasonable request.
