# Supplementary material for: mTOR pathway mediates endoplasmic reticulum stress-induced CD4+ T cell apoptosis in septic mice
Source: Apoptosis. 2022 Jun 27;27(9-10):740–50. doi: 10.1007/s10495-022-01740-1 (PMC9482898; doi:10.1007/s10495-022-01740-1)
Supplement: Supplementary file 1 — Supplementary file1 (DOCX 1439 kb) [file 10495_2022_1740_MOESM1_ESM.docx]

**
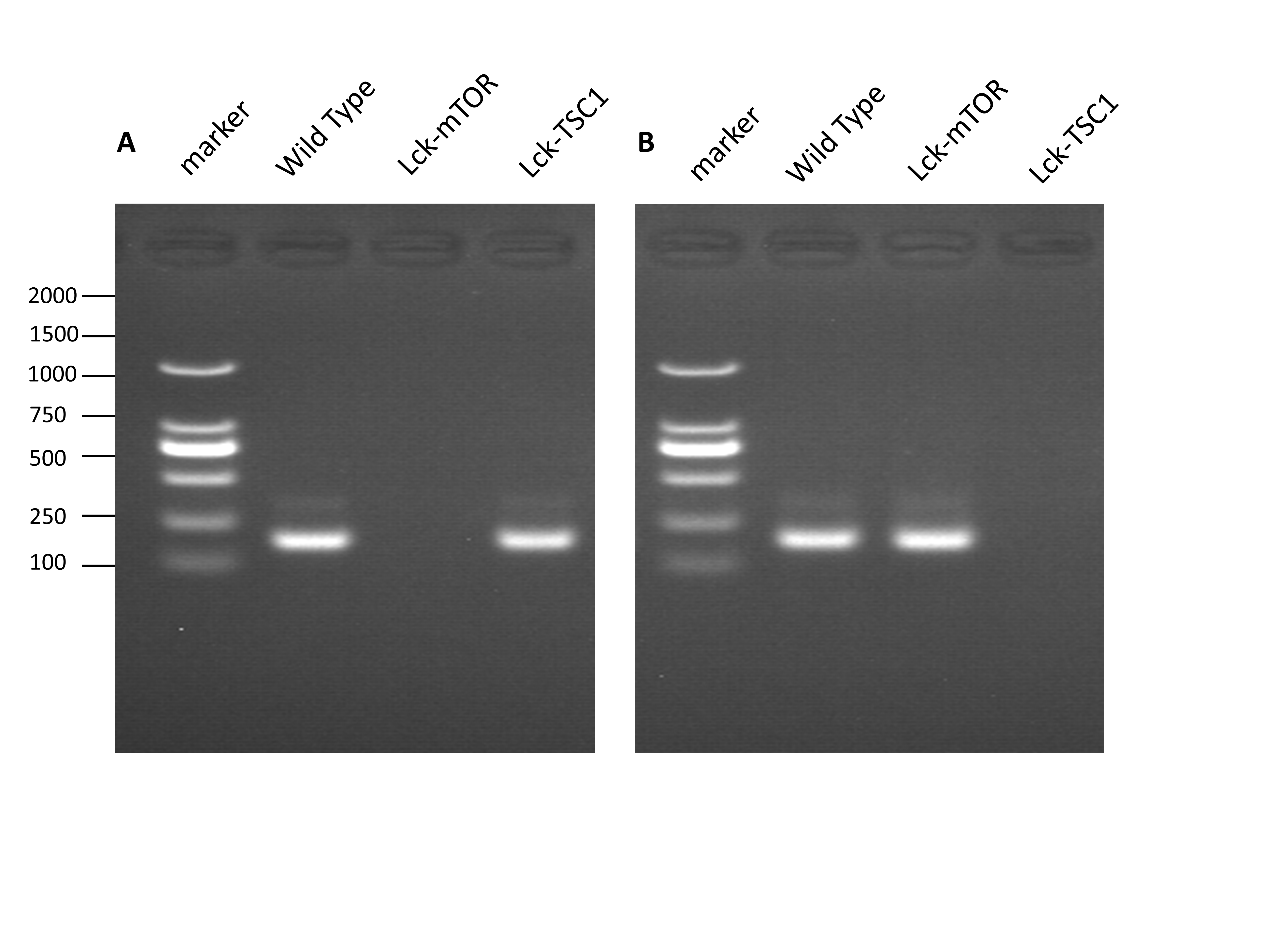
**

**Figure S1. Gene identification of T cell-specific knockout *mTOR*/*TSC1* mice.** The mRNA expression levels of *mTOR* (A) and *TSC1* (B) in mice were detected by RT-PCR to confirm knockout of the target genes.


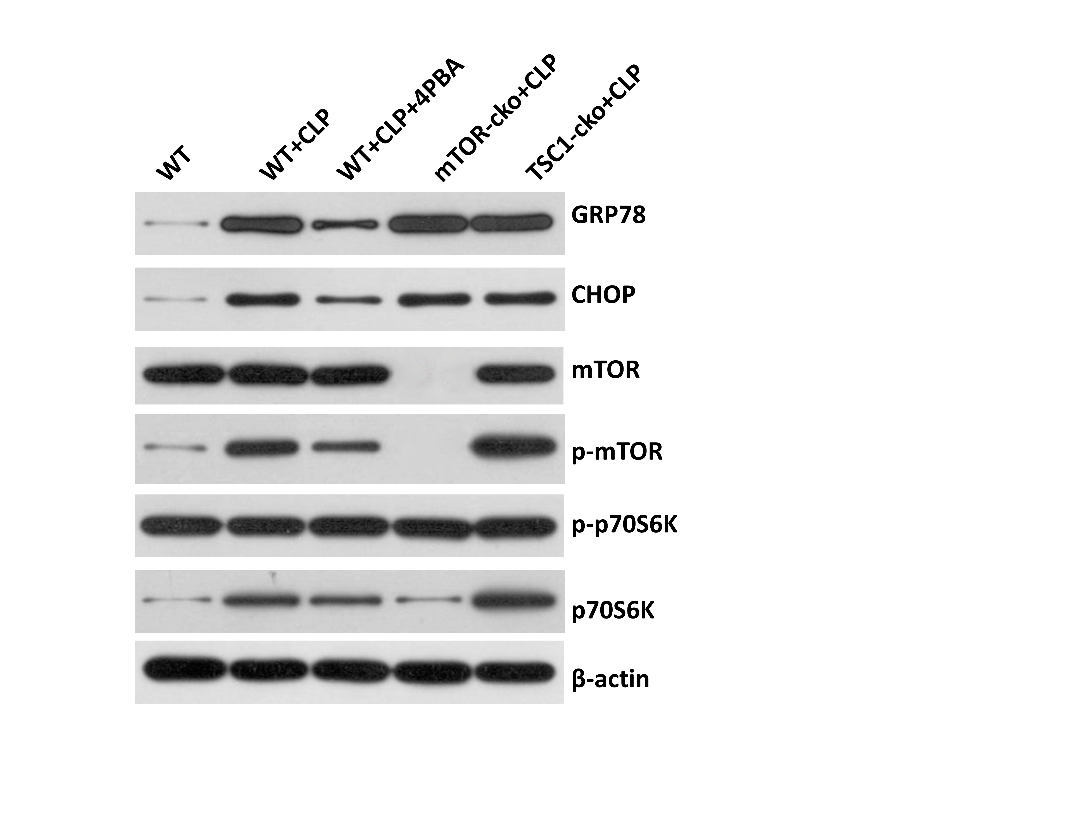

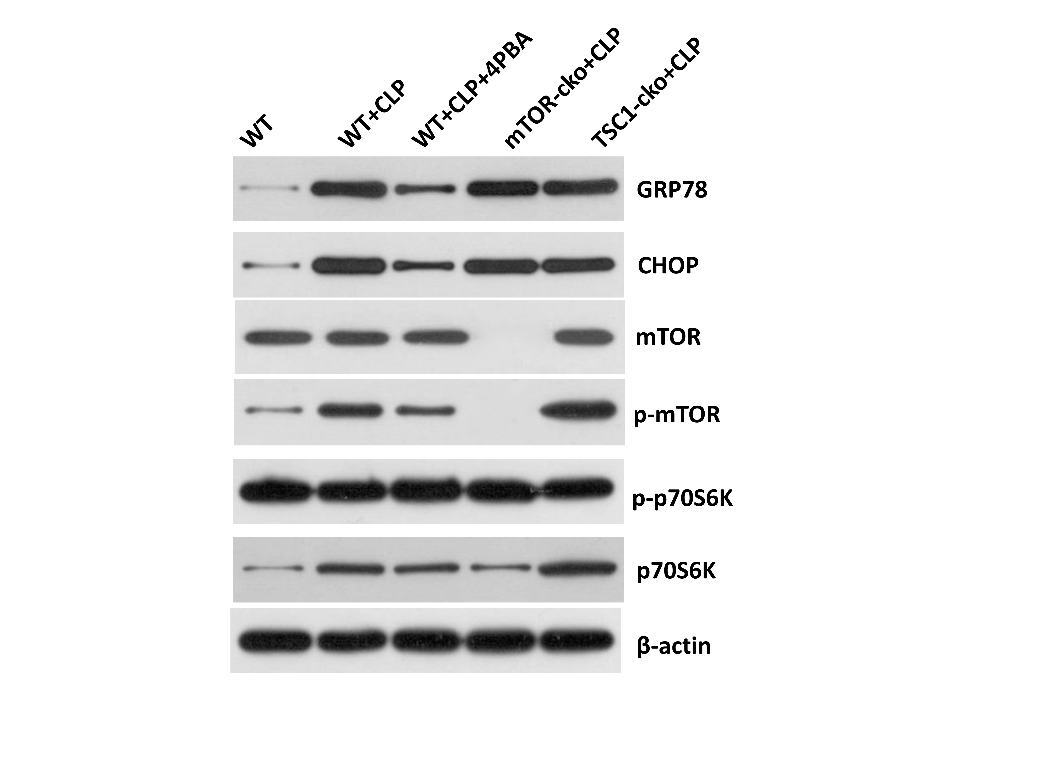
**WB figures of the other two repeated experiments.**

**Figures S2-3: Repeated WB results of Fig 4.**


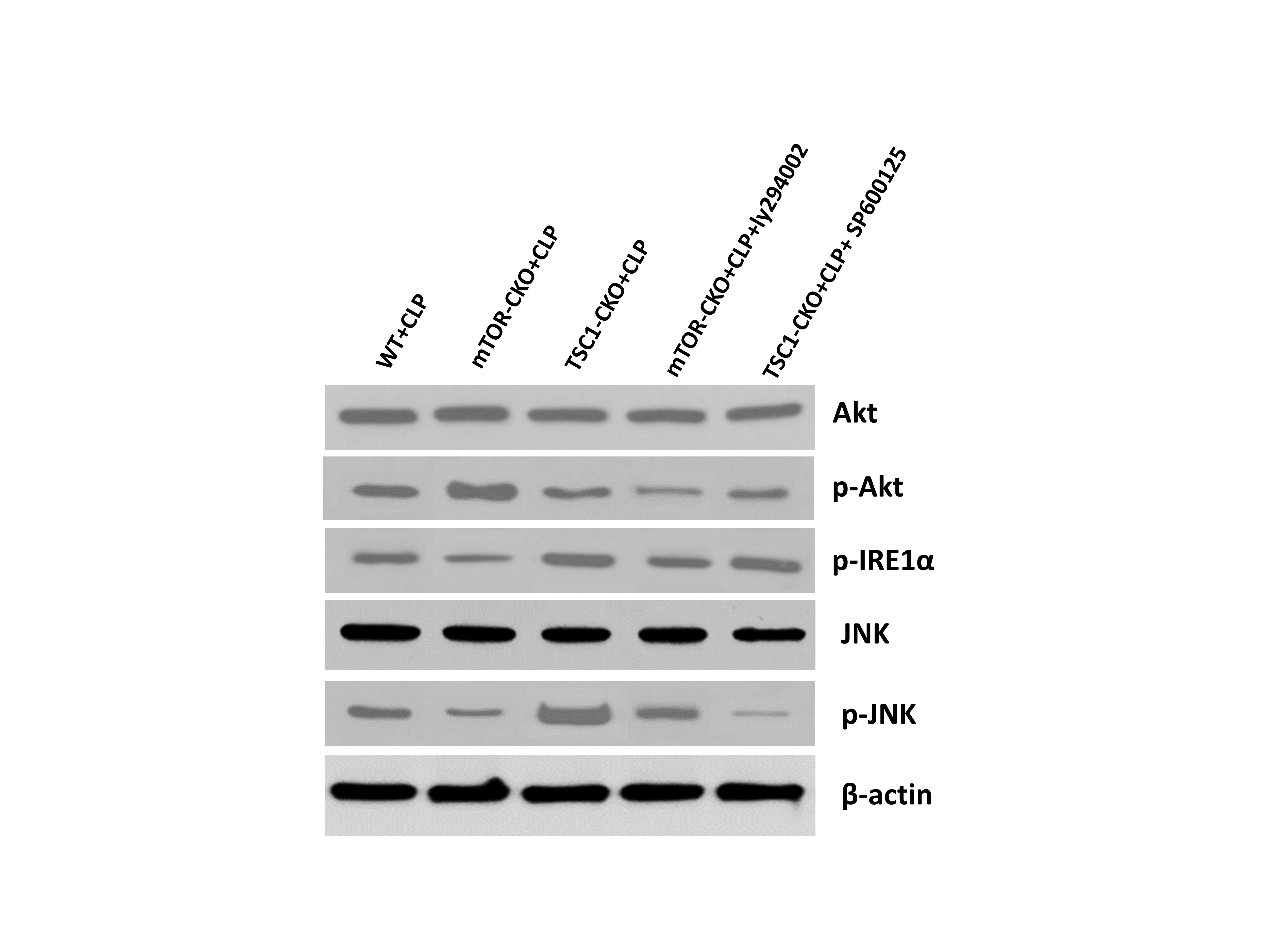

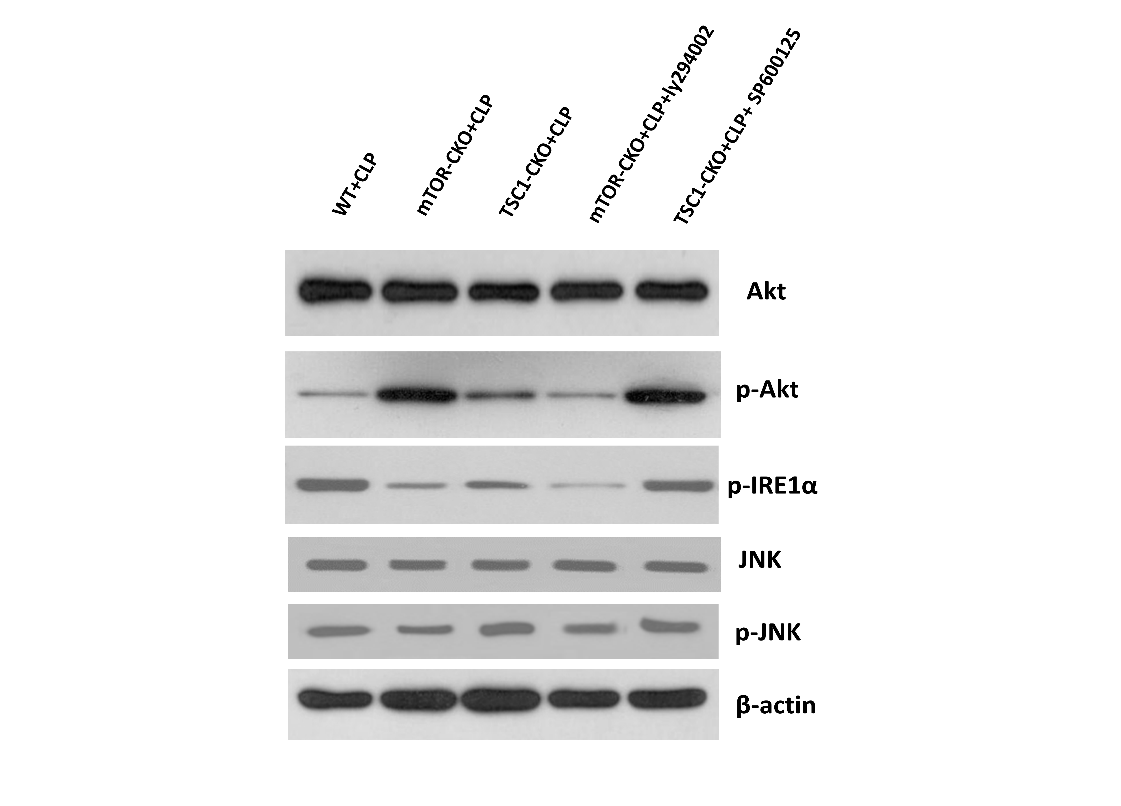


**Figures S4-5: Repeated WB results of Fig 5.**


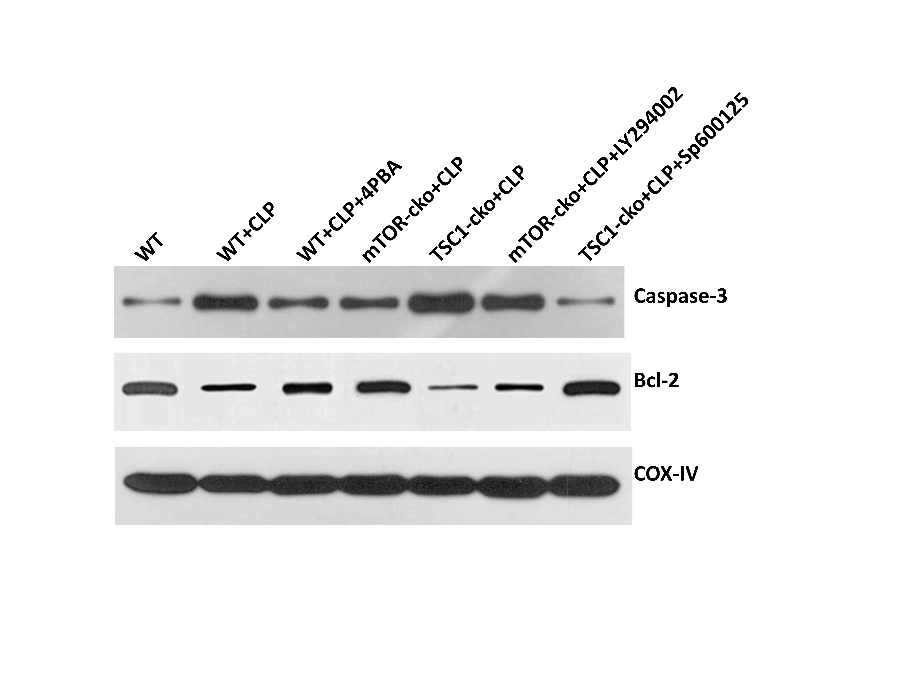

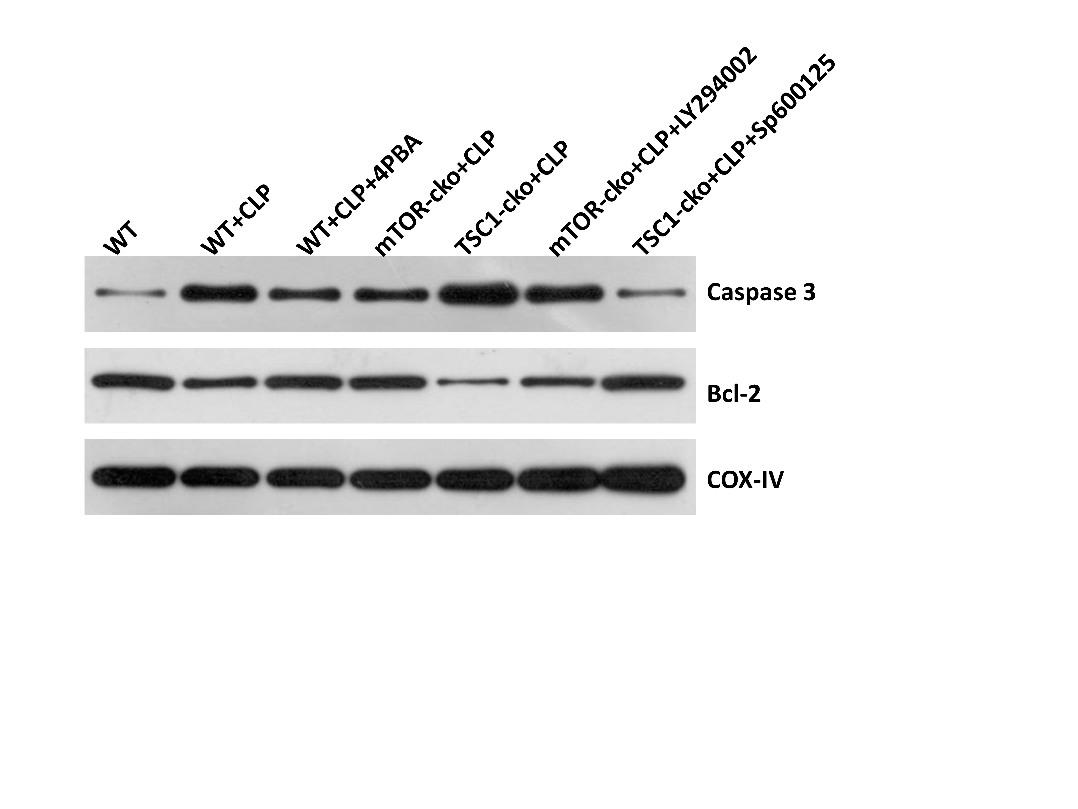


**Figures S6-7: Repeated WB results of Fig 6.**
